# Manufacturing Technics for Fabric/Fiber-Based Triboelectric Nanogenerators: From Yarns to Micro-Nanofibers

**DOI:** 10.3390/nano12152703

**Published:** 2022-08-05

**Authors:** Chonghui Fan, Yuxin Zhang, Shiqin Liao, Min Zhao, Pengfei Lv, Qufu Wei

**Affiliations:** 1 Key Laboratory of Eco-Textiles, Ministry of Education, Jiangnan University, Wuxi 214122, China; 2 Jiangxi Centre for Modern Apparel Engineering and Technology, Jiangxi Institute of Fashion Technology, Nanchang 330201, China; 3 College of Textile and Clothing, Nantong University, Nantong 226019, China

**Keywords:** triboelectric nanogenerator, fabric/fiber structure, wearable self-powered sensors

## Abstract

Triboelectric nanogenerator (TENG), as a green energy harvesting technology, has aroused tremendous interest across many fields, such as wearable electronics, implanted electronic devices, and human-machine interfaces. Fabric and fiber-structured materials are excellent candidates for TENG materials due to their inherent flexibility, low cost, and high wearing comfort. Consequently, it is crucial to combine TENG with fabric/fiber materials to simultaneously leverage their mechanical energy harvesting and wearability advantages. In this review, the structure and fundamentals of TENG are briefly explained, followed by the introduction of three distinct methods for preparing fabric/fiber structures: spinning and weaving, wet spinning, and electrospinning. In the meantime, their applications have been discussed, focusing primarily on energy harvesting and wearable self-powered sensors. Finally, we discussed the future and challenges of fabric and fiber-based TENGs.

## 1. Introduction

In recent years, mobile wearable devices and wireless communication networks, as symbols of the new era, have gradually changed people’s ways of life [1,2,3]. Particularly, flexible energy supply devices have attracted extensive attention with the development of wearable electronic markets. Although the external power supplies of wearable electronic products, such as small solid batteries, supercapacitors, lithium-ion batteries, etc., are becoming more and more miniaturized and portable, their rigid nature contradicts the flexibility of fabric, resulting in inferior comfort of wear [4,5]. In addition, these battery devices cannot provide a long-term energy supply during repeated charging/discharging. Moreover, a large amount of wasted electronics needs high disposal and recycling costs during the disposal and recycling process, which also lead to soil and water pollution [6,7,8]. What is more dangerous is that in complex working environments, they might easily cause leakage of electrolyte and oxidized materials within the battery, resulting in severe harm to users. Therefore, from the perspective of economic and sustainable development, rigid battery devices are a poor choice for the future development of wearable sensors [9,10]. Hence, it is of the utmost importance to select a sustainable green and environmentally friendly flexible power supply that is not only integrated and miniaturized, but also environmentally credible and will not accidentally harm the human body [11,12,13,14].

Collecting energy from the surrounding environment is one potential solution to this issue. As a revolutionary energy harvesting technology, triboelectric nanogenerators (TENGs) utilize simple construction and mechanisms to effectively harvest mechanical energy and play a crucial role in the construction of portable power sources or self-powered systems [15,16,17]. Moreover, compared to traditional power supply systems, TENGs have the advantages of light weight, simple structure, and strong material adaptability [18,19]. As one of the most abundant energies in the human living environment, mechanical energy possesses the qualities of continuity, independence, easy access, and widespread existence. It has significant application potential in the fields of smart wearables and biomedical engineering. As the human body is not only a rich source of green mechanical energy but also an application terminal for smart wearables, the acquisition and utilization of required energy can be accomplished through human movement by integrating nanogenerator and human movement [20,21]. Although some polymer films (e.g., polytetrafluoroethylene, polydimethylsiloxane) can also adhere to the human body (e.g., knees, arms) to harvest mechanical energy or monitor movements, the poor wearing comfort and durability remains a great challenge. By comparison, fabrics and fiber materials have natural wearability, inherent durability, and can withstand complex mechanical deformations such as stretch, twist, bend and tear, owning to its uniqueness. Thus, the integration of cutting-edge energy collection technology and fabric/fiber materials are advantageous for the development of wearable self-powered sensor [22,23].

**Figure 1:**
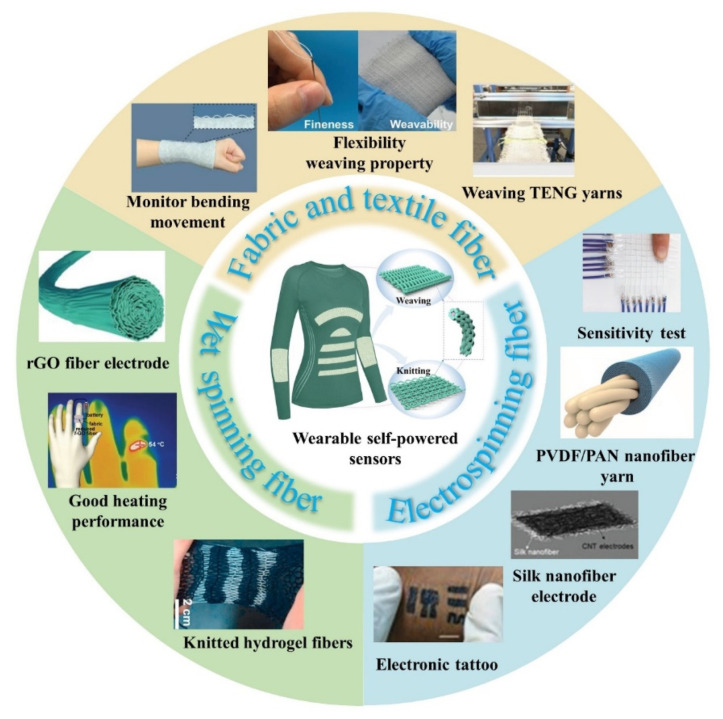
Schematic of recently reported fabric/fiber-based TENG.

This review summarizes the application advantages of fabric/fiber material in the fields of TENG and the feasibility of self-powered wearable sensors. Firstly, the structure design and operating mechanisms of TENG is introduced. Followed by the uniqueness of fabric/fiber materials based on TENG. Fabric/fiber materials are divided into three categories based on their preparation methods: fabric and textile fiber (spinning and weaving method), wet spinning fiber materials (wet spinning method), and electrospinning nanofibers (electrospun method). Figure 1 depicts recent research progress of fabric/fiber-based TENGs made mainly through these methods [24,25,26,27,28]. In addition to energy harvesting, human motion detection and functional applications can also be incorporated. These applications fully confirm the vast potential of fabric/fiber-based TENG in the field of self-powered wearable sensors, bringing revolutionary changes to the future development of wearable technology and greatly promoting the robust growth of the wearable electronics market. It is hoped that this review can bring new enlightenment to the researchers and promote more research results in the field of fabric/fiber-based TENG.

## 2. The Mechanism and Structure Design of TENG

**Figure 2:**
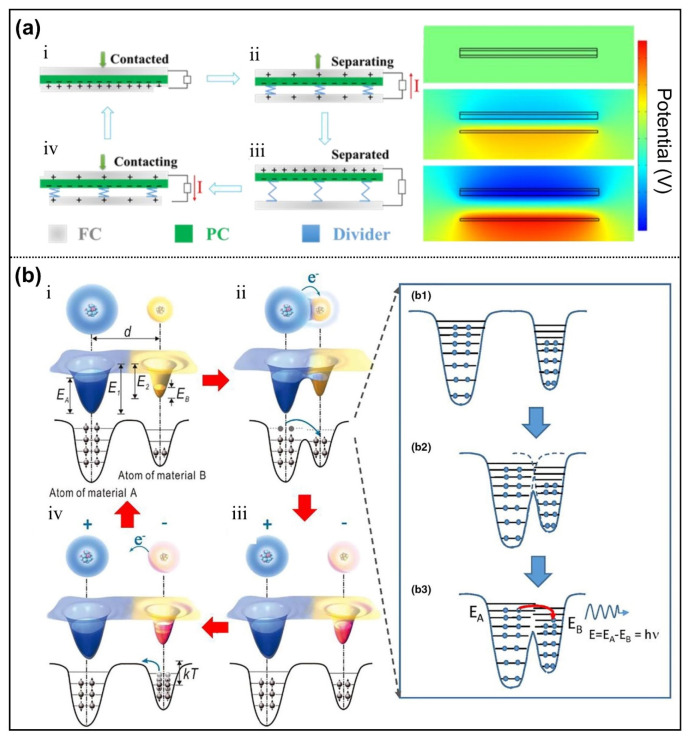
Working mechanism of TENG. (**a**) Working models, which include: (i) original state, (ii) separating state, (iii) fully separated, (iv) contacting each other. Reprinted with permission from Ref. [35]. Copyright 2020 American Chemical Society. (**b**) The electron cloud/potential well mechanism for the contact-electrification phenomenon. Reprinted with permission from Ref. [31]. Copyright 2022 Elsevier.

TENG is a new energy collection technology device proposed by Professor Wang in 2012 [29,30]. It can convert surrounding mechanical energy into electric energy by utilizing the dual effects of contact electrification (CE) and electrostatic induction (EI). Specifically, when two kinds of materials are in contact with each other under external force (Figure 2a(i),(iv)), the surface of the materials will produce positive or negative charges due to different polarities of triboelectric materials. When the two materials are separated (Figure 2a(ii),(iii)), the positive and negative static charges generated by contact electric charge are also detached, thus the induced potential difference is correspondingly produced on electrodes. If the two electrodes are in loaded or in a short-circuit state, the electrons driven by potential difference would flow in the external circuits, thus transform mechanic energy into electric energy. The difference in surface potential significantly affects the triboelectric property. In short, this CE phenomenon is closely related to electron transfer, which can be easily explained by the electron cloud/potential well mechanism (Figure 2b) [31]. Compared to piezoelectric nanogenerators (PENG) and pyroelectric nanogenerators, TENG have the advantages of high output power, a wide selection of materials, simple fabrication, low cost, and light weight [32,33,34]. In the past decade, TENG has attracted significant attention, and a number of research advancements have been accomplished in terms of device working mode design, physical mechanism disclosure, and study of practical applications.

**Figure 3:**
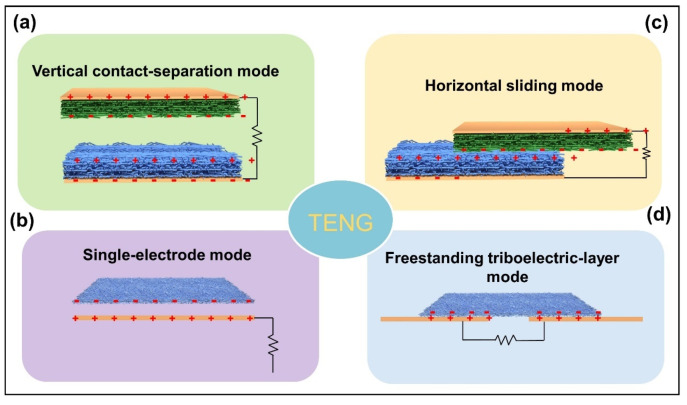
The basic structure mode of TENGs. (**a**) Vertical contact-separation model. (**b**) Single-electrode mode. (**c**) Horizontal sliding mode. (**d**) Freestanding triboelectric-layer mode.

In particular, according to the polarization direction and electrode structure, as shown in Figure 3, the TENG can be mainly divided into four basic working modes: vertical contact-separation mode, single-electrode mode, horizontal sliding mode and freestanding triboelectric-layer mode [36,37]. Based on the different working modes of TENG, various forms of mechanical energy collection and self-powered sensors in various application scenarios can be realized. The basic working modes are as follows:

### 2.1. Vertical Contact-Separation Mode

The vertical contact-separation model is the simplest and most widely studied of TENGs, with the highest instantaneous power output. Its typical structure consists of two kinds of dielectric films with electrodes assembled vertically on the back (Figure 3a). The working mechanisms are similar to what is depicted in Figure 2a. When the dielectric films make recontact, the potential difference caused by triboelectric charges disappears, and electrons flow in the opposite direction due to the dual effects of CE and EI [36]. Under the periodic external force, the vertical contact and separation between two triboelectric layers of TENG generate the periodic alternating current (AC).

### 2.2. Single-Electrode Mode

In daily life, it is inconvenient to attach electrodes onto the surface of triboelectric materials, especially when two moving materials contact each other, which would lead to uncertainty. To solve this problem, researchers proposed a single-electrode mode TENG with only one electrode attached onto the dielectric film, as shown in Figure 3b. In this way, only the chosen material is fixed with an electrode. The opposite dielectric material can be any other moving material, and can even be used as electrode [38]. However, due to the electrostatic shielding, the overall electric output of the single-electrode TENG is only half of the dual-electrodes mode. However, the advantage of single-electrode TENG affords it a wide range of application prospects in movement monitoring and energy collection of water droplets, intelligent sensing, human-computer interaction and other fields [39,40].

### 2.3. Horizontal Sliding Mode

The initial structure of the horizontal sliding mode TENG is similar to that of the vertical contact-separation mode, but the films of the two friction materials remain in close contact (Figure 3c). Driven by external forces, the two friction materials slip horizontally which is parallel to the surface and generate friction charges on the surface. The lateral separation of the center of the friction charge forms a potential difference that drives electrons through the external load to balance the electrostatic field of the friction charge. The horizontal sliding mode TENG is separated and closed by periodic sliding of two friction materials in the horizontal direction, producing AC output. However, the sliding friction between solid materials severely wears the materials and generates heat, which reduces the efficiency and durability of TENG [41].

### 2.4. Freestanding Triboelectric-Layer Mode

Freestanding triboelectric-layer mode TENG usually composed of an independent friction layer and a fixed electrode (Figure 3d), the periodical movement of the friction layer between two electrodes causes the potential difference, so as to drive the electron to reciprocate between two electrodes through circuits in order to equilibrate the changes of potential difference [42]. Similar to single-electrode TENG structure, the freestanding mode is simple in design and fabrication, besides, the independent friction layer can move periodically without restriction and is not affected by electrostatic shielding effect, so it has higher energy conversion efficiency.

## 3. Fabric/Fiber-Based Triboelectric Layers in TENGs

Fabric/fiber-based TENG is a new type of intelligent self-powered wearable device prepared by using fabric/fiber material or processing technology and combined with the principle of nanogenerator [43,44]. Integrated the advanced mechanical energy harvesting technology into fabric/fiber materials or textile structures, giving full play to the dual advantages of fabric/fiber and TENG. Nanogenerator technology endows fabric/fiber materials with the ability of mechanical energy collection and self-powered multifunctional sensing, while fabric/fiber materials provide a multifunctional structural carrier and unique flexible wearable feasibility for the nanogenerator [38].

In this section, we discussed the different preparation methods of fabric/fiber materials for TENG, which are mainly divided into weaving, wet spinning and electrospinning methods. The summary and comparison of components, manufacture methods and triboelectric outputs of these fabric/fiber-based TENGs are shown in Table 1. Different weaving processes enrich the preparation methods of fabric/fiber-based TENG and lay the foundation for its large-scale production. On one hand, nanogenerators can be easily integrated with fabric or fiber materials or designed into fabric or fiber structures, giving fabric or fiber the ability of mechanical energy collection and self-powered sensing. On the other hand, the good wear resistance, permeability, comfort, structural flexibility, and low cost of textiles provide good conditions for the wide application of nanogenerators. Because human kinetic energy is associated with human motion, which is an inexhaustible green energy without cost, and clothing is an indispensable necessity in people’s lives, energy can be easily collected from human body movement by combining clothing and TENG, which can also be a highly attractive self-powered platform for personal wearable.

### 3.1. Fabric and Textile Fiber-Based TENG

It is well known that the primary structure of TENG consists of a friction layer, an electrode layer, and a wire, with the choosing of the friction layer and electrode layer being crucial for the flexible wearable application of TENG. Traditional electrodes, on the other hand, consisted mostly of non-flexible metals adhered to the friction layer’s surface, resulting in poor wearing comfort. In addition, metal electrodes and friction materials also have a tendency to wear off in severe exterior settings, which is unsustainable for the steady and long-term storage of human mechanical energy.

As a traditional textile preparation method, spinning and weaving process has a long history and mature preparation experience, and has been adopted by many researchers. Weaving allows for the production of woven textiles, knitted fabrics, and even three-dimensional (3D) spacer fabrics. In order to overcome the incompatibility between the flexible friction layer and the rigid electrode and enhance the energy harvesting efficiency [58,59], numerous researchers have adopted flexible fabric/fiber materials and used as flexible electrode and friction layer by spinning and weaving process through a series of modifications.

**Figure 4:**
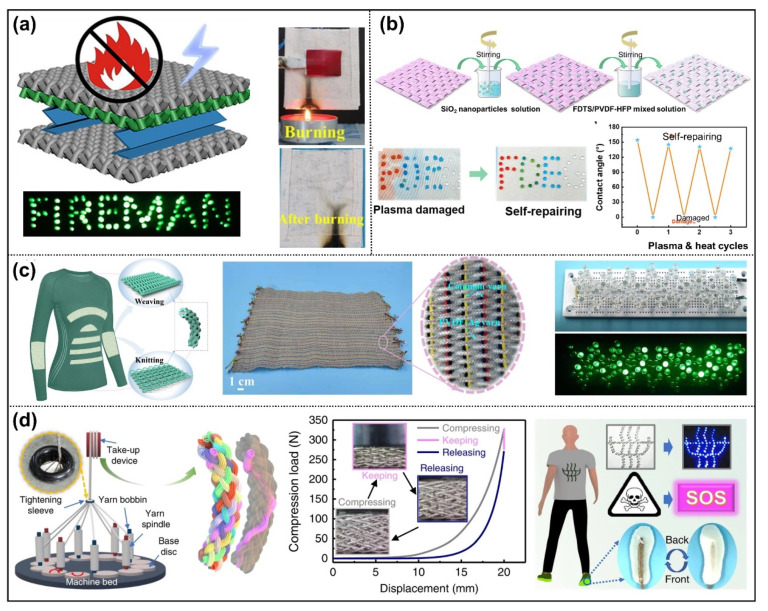
Fabric and yarn structured TENG. (**a**) A flame-retardant fabric coated with silver paste and PTFE as electrode and triboelectric layers. Reprinted with permission from Ref. [35]. Copyright 2020 American Chemical Society. (**b**) A kind of hydrophobic and self-cleaning fabric coated with fluorinated polymer and SiO_2_ particles for water droplet energy harvesting. Reprinted with permission from Ref. [45]. Copyright 2021 American Chemical Society. (**c**) A large-scale core-shell type knitting triboelectric fiber by weaving PVDF yarns onto silver-coated nylon yarns. Reprinted with permission from Ref. [46]. Copyright 2022 Wiley. (**d**) A 3D braided intelligent self-powered and sensing fabric based on the shape adjustable structure with high compression rebound feature. Reprinted with permission from Ref. [48]. Copyright 2020 Nature.

Chen et al. prepared a flame-retardant cotton as the substrate, and subsequently coated it with silver paste as conductive electrode with flame-retardancy [35]. In addition, polytetrafluoroethylene (PTFE) emulsion was sprayed to cotton fabric and employed as an electronegative friction layer in conjunction with the modified cotton layer for flame-retardant TENG (Figure 4a). In particular, the TENG maintained 49.2% of the original electric performance after burning at 17 various locations. In contrast, Ye et al. [45] directly employed conductive fabric as an electrode and hydrophobic polyester fabric as a friction layer to create a self-cleaning, single-electrode TENG. Silicon oxide (SiO_2_) nanoparticles, perfluorodecyltrichlor osilane (FDTS), and poly(vinylidenefluoride-co-hexafluoropropylene) (PVDF-HFP) are dipped onto the fabric’s surface to impart hydrophobicity and resilience to acids and alkalis (Figure 4b). In addition, plasma-damaged hydrophobic fabric may be restored cyclically because PVDF-HFP can facilitate the movement of hydrophobic FDTS molecules to the surface of the fiber under heating conditions, therefore accomplishing the hydrophobic self-repair function. In this study, the TENG fabric was shown to be machinable, comfortable to wear, and functionally finished for self-powered sensing applications.

Currently, the vast majority of TENG are produced by coating or adhering a layer of dielectric material to the surface of the electrode [59,60], or by spraying conductive material (carbon nanotubes (CNTs), reduced graphene oxide (rGO), carbon black (CB), or Ag nanowires) directly on the surface of the friction layer, which drastically reduces permeability and wear comfort. Therefore, it is crucial to choose an electrode material with superior conductivity, flexibility, and mechanical strength. In order to solve the above problem, conductive yarns, such as silver-plated yarns, stainless steel yarns, and NiCu yarns, have recently been used as the electrode. Thus, the friction materials can be wrapped on the surface of conductive yarns by textile technology to form integrated core-shell structure TENG yarns. The related study was recently presented. For example, Dong et al. reported a large-scale prepared core-shell type knitting triboelectric fiber [46]. As depicted in Figure 4c, silver-coated nylon yarns were chosen as the electrode for its good flexibility, stitchability and excellent conductivity. By weaving polyvinylidene fluoride (PVDF) yarns onto the surface of silver-coated nylon yarns, uniformly thick triboelectric fibers with a continuous preparation process were created. Triboelectric yarns can be woven into a desired knit fabric region. Benefiting from the structure integrity, the TENG fiber exhibits a solid structure, high tensile strength, and excellent electrical output performance (light LED bulbs, power density of 1008 mW m^−2^), in addition to being washable. More importantly, this independent triboelectric fiber can be further integrated into smart fabrics of different fabric structures for energy collection and self-actuated sensing functions. More recently, flame retardant polyimide (PI) yarn has also been used to prepare core-shell structure friction materials. Ma et al. engineered a 3D honeycomb structure based on PI winding yarn for TENG woven fabric [47]. The flame-retardant PI fiber was wrapped around the surface of conductive yarn by continuous hollow spindle fancy twisting machine technology. The multi-functional 3D honeycomb knit fabric prepared by the structure design not only has excellent flame retardancy and rescue positioning function, but also gives the fabric excellent noise reduction ability. In order to improve the output properties of fabric-based TENG, researchers have designed and optimized different textile structures. Wang’s group proposed a 3D braided intelligent self-powered and sensing fabric based on the shape-adjustable structure with a high compression rebound feature (Figure 4d) [48]. The 3D braided self-powered and sensing fabric is composed of an outer braided support frame and an inner shaft core column, which enables fast recovery under load and unloading with the benefits of high compression resilience (compression rebound coefficient: 60%), a variety of sectional shape designs (rectangular, square, circular, etc.), and increased power output (peak power density: 26 W m^−3^). This intelligent yarn sewn onto insoles is capable of sending rescue signals on demand due to its rapid sensitivity to micro pressures and vibration energy collection capabilities.

### 3.2. Micron Fiber-Based TENG Prepared by Wet Spinning

**Figure 5:**
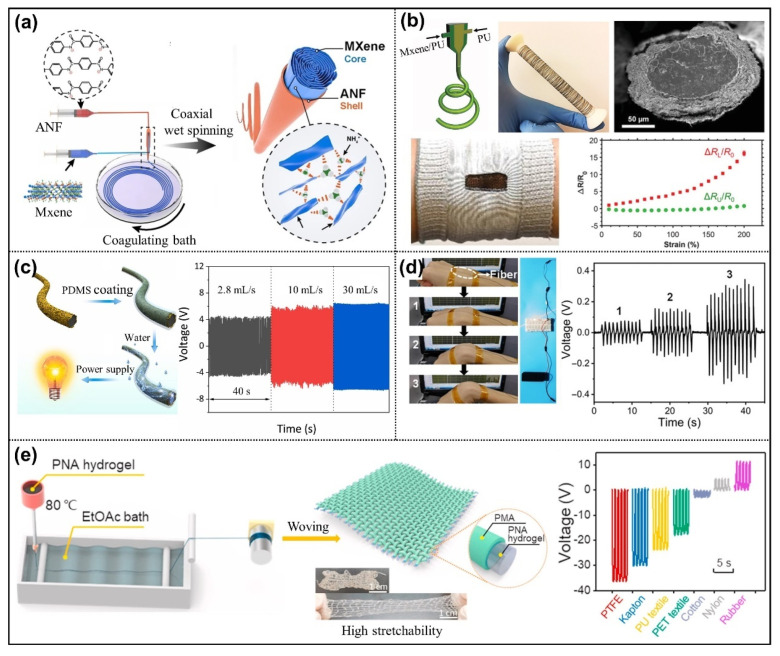
Preparation of micron fiber-based electrode materials applied for energy harvesting by wet spinning method. (**a**) Tough and stable aramid nanofiber/Mxene conductive coaxial fibers. Reprinted with permission from Ref. [62]. Copyright 2022 Springer. (**b**) A highly stretchable Mxene/polyurethane composite polymer fibers with strain sensing properties. Reprinted with permission from Ref. [64]. Copyright 2022 Wiley-VCH. (**c**) High electrical conductivity and tensile strength elastomer fibers by coating PDMS layer for water droplet energy harvesting. Reprinted with permission from Ref. [49]. Copyright 2021 Elsevier. (**d**) Conductance-stable, liquid metal sheath-core microfibers for monitoring human activities. Reprinted with permission from Ref. [66]. Copyright 2021 Science. (**e**) The preparation of stretchable, conductive and self-healing core-sheath hydrogel fibers for wearable electronics. Reprinted with permission from Ref. [28]. Copyright 2020 Elsevier.

Wet spinning technology is a kind of fiber spinning method in which a polymer is dissolved in a solvent and sprayed through a spinneret into a coagulation bath to form fibers. Wet spinning has been widely studied for fabricating 1D fiber-based electronic devices in recent years because of its continuity, stability and large-scale production. In this manner, not only can stretchable and flexible polymer fibers be manufactured, but also conductive polymers and materials may be successfully incorporated into fibers to provide both high conductivity and stretchability. Thus, due to its good conductivity, braided ability and light weight, the 1D conductive fiber can be used as a flexible electrode by researchers and applied in sensing fields, such as pressure sensors, strain sensors, and TENGs. The precursor solution of conductive fibers is usually composed of polymers and conductive fillers. Polymers such as thermoplastic polyurethane (TPU), poly (styrene-butadiene styrene) (SBS) etc., can not only improve the stretch property of the conductive fiber but also endow the fiber with good strength including some enhanced nanofibers. (Figure 5a) [61,62]. Typically, conductive fillers consist of inorganic compounds such as CNT, CB, rGO, and Mxene, conductive polymers (polyaniline, polyacetylene, polypyrylene), and metal components (liquid metal). For the durability and conductivity of cured fibers as a flexible electrode material, the interfacial bond and compatibility between polymers and conductive fillers are crucial.

Although some conductive materials may be used directly in wet spinning to make fibers, their inherent brittleness and low mechanical qualities restrict their uses. As a result, the incorporation of polymers such as nanofibers can substantially enhance the strength performance of conductive composite substrates. He et al. used polyamide nanofibers as functional additives for improving the strength of Mxene fibers. The mechanical strength of composite fibers reached 104 MPa [63]. Gogotsi et al. fabricated a composite fiber using wet spinning technology with good braided, high electrical conductivity and stretchability that can be used in wearable tensile strain sensors [64]. In the system, Mxene as a conductive filler, PU as a substrate, and acetic acid (AcOH) as the condensation bath are used to realize the control of spinning with Mxene content in the range of 0–100 wt%. Moreover, the Mxene/PU composite fiber was woven into the fabric by weft knitting machine as shown in Figure 5b. The sensitivity of the fabric is significantly higher than that of the traditional knitted fabric. It provides a practical platform for a series of wearable applications requiring movement monitoring such as remote health monitoring for patients and exercise guidance. In addition to a single conductive filler, a variety of conductive materials can be added in wet spinning precursors to improve the conductive properties of fibers in a high tensile state. For example, by uniformly dispersing 1D CNT and 2D Mxene in a PU solution [49], the prepared fiber composites could not only enhance the mechanical properties, but also maintain the electrical conductivity under stretching. When the fiber is strained, Mxene can be used as a bridge to connect the CNT and maintain the sliding of the CNT. Due to the good electrical conductivity and tensile strength, an enhanced flexible fiber-based TENG can be obtained by further coating polydimethylsiloxane (PDMS) as a triboelectric layer used as an effective water droplet energy harvesting device. It shows that the generated voltage reached 6 V at a 30 mL s^−1^ flow rate (Figure 5c).

In addition to carbon-based materials, liquid metals have been widely utilized in the sensing field due to their unparalleled conductivity and mobility. Directly depositing the liquid metal on the fiber substrate would readily produce interface bonding issues because to the high surface tension. Furthermore, contact to the oxygen atmosphere may generate an oxide layer on the surface of the liquid metal, which then greatly hampers conductivity. To solve this problem, wrapping the liquid metal can isolate external surroundings without affecting its electrical property by wet spinning technology [65], especially due to its good applicability and compatibility with metal nanoparticles and polymers. A group led by Wu devised a three-layer coaxial wet spinning technology that can manufacture liquid metal sheath-core fibers with outstanding conductivity and tension-insensitive resistance [66]. As seen in Figure 5d, the initial conductivity is as high as 4.35 × 10^4^ S m^−1^, which can easily light a bulb even in 55 cm length. The fiber sheath is composed of fluorine-containing elastomer, whereas the core layer is composed of the same elastomer with liquid metal nanoparticles. The sheath-core structure and dipole interaction between fluorine-containing elastomer and liquid metal oxide layer cause the conductive path of liquid metal particles in the core layer to undergo reversible conformal deformation upon stretching, allowing for high tensile property and resistance stability without liquid metal leakage concerns. Due to its stable conductivity and unique structure, the prepared elastic liquid metal sheath-core fiber can also be used as self-powered sensors. The liquid metal in the inner layer was used as an effective electrode material due to its excellent conductivity, while the outer layer’s fluorinated polymer is used as a friction material due to its high tribonegativity. In this way, different perceived voltage signals can be detected by bending the wrist at different angles. In addition to the polymer fibers mentioned above, hydrogels, as soft 3D network structure substrates [67], are often used as flexible electrodes for stretchable sensors, electroluminescence, TENG, and other sensing fields due to their unique characteristics, including high tensile strength, ionic conductivity, and compatibility [68,69]. However, hydrogels prepared by one-pot polymerization methods are mainly 2D membrane materials, which severely restricts their braidability and wearability. Additionally, long-term exposure to the environment can easily cause dehydration and evaporation problems, resulting in reduced ionic conductivity, which seriously affects their application prospects in sensing fields. At present, hydrogel fibers prepared by wet spinning have been used as ionic electrodes for TENG [28]. In order to prevent water evaporation, the surface of hydrogel fiber was further coated with polymethyl acrylate (PMA), which ensured the long-term stability of hydrogel electrode. First, the hydrogel fiber resulting from the physical cross-linking copolymerization of acrylamide and acryloylglycine shows excellent mechanical strength (2.27 MPa), strain properties (900%), electrical conductivity (0.50 S m^−1^) and self-healing capabilities (Figure 5e). The PMA coating may then be utilized as a friction material in conjunction with a hydrogel electrode for TENG applications. Intriguingly, the high-strength hydrogel fibers produced by wet spinning can be woven into fibrous fabrics as flexible TENG devices for large-scale production. These devices convert mechanical motion energy into electrical energy, offering great potential for the next generation of multifunctional smart textiles and wearable electronics.

### 3.3. Nanofiber-Based TENG Prepared by Electrospinning

As one of the methods of preparing micro/nano materials, electrospinning technology has been widely adopted for its advantages of simple operation, low cost and continuous production. Specifically, the nanofiber membrane obtained by electrospinning has a large specific surface area and is also breathable. Moreover, the size of the fiber and various structures such as beaded fibers, hollow fibers and core-shell fibers can be controlled by adjusting different spinning parameters. Compared with the membrane materials fabricated by spin-coating and dipping-coating methods, the electrospinning nanofiber membrane can enhance the output performance of TENG due to its large effective friction area. Therefore, many researchers have carried out a lot of studies on the application of electrospinning in TENG.

**Figure 6:**
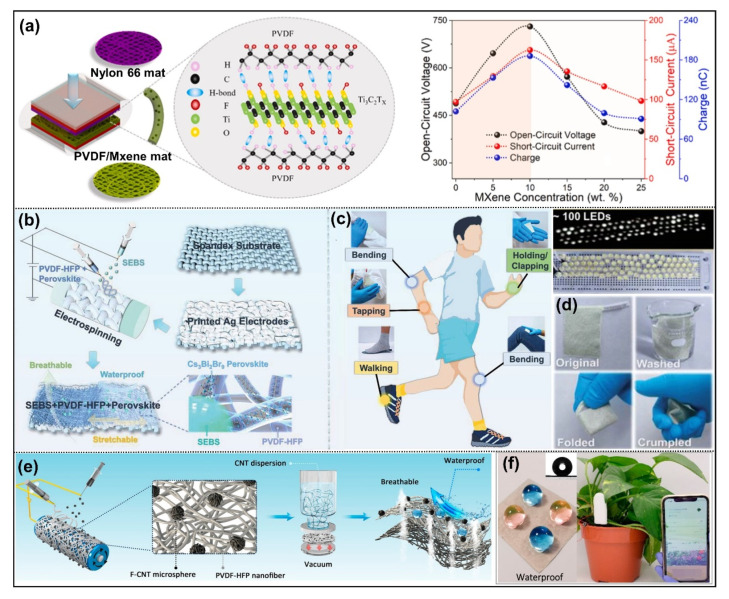
Nanofiber based triboelectric materials applied for energy harvesting prepared by electrospinning. (**a**) The structure of PVDF/Mxene nanofiber for TENG. Reprinted with permission from Ref. [51]. Copyright 2021 Elsevier. (**b**) A stretchable and washable perovskite/PVDF-HFP nanofiber composite. (**c**) Human movements monitoring and mechanical energy collection when people bend their arms and walk. (**d**) Good washing stability. Reprinted with permission from Ref. [53]. Copyright 2022 Wiley. (**e**) A waterproof and breathable TENG nanofiber composite for environmental energy harvesting. (**f**) Actual application for plant leaves detection. Reprinted with permission from Ref. [54]. Copyright 2021 American Chemical Society.

Due to its good spinnability and strong electronegativity, β-phase PVDF is mostly used as a tribonegative material. In recent years, researchers have found that the tribological properties of PVDF nanofibers can be greatly enhanced by adding inorganic materials such as carbon materials, metallic oxide and metal nanoparticles, etc,. [44,50,70]. The addition of these fillers is mainly to functionalize the triboelectric materials and improve their charge capture ability. For example, Shi et al. showed a PVDF/Graphene membrane prepared by spin-coating and electrospinning methods as the negative friction layer [44]. The results show that the nanofibers have a higher triboelectric output than the spin-coating films and the addition of graphene greatly improved the triboelectric performance. This is due to graphene’s ability to improve the surface potential and charge capture ability of PVDF, thereby promoting the formation of high crystalline β-phase PVDF and thereby significantly improving the total transfer charge density of TENGs. In addition, Mxene, as a new kind of 2D transition metal carbide and nitride, can also be used as effective polymer fillers due to their inherent high electronegativity and good electrical conductivity [51]. The dielectric constant and surface charge density of PVDF nanofibers were increased by 270% and 80% by introducing conductive Mxene nanosheets (Figure 6a), while an excess of Mxene (more than 10 wt%) could result in decreased output performance. Furthermore, some metals and metal oxides can accelerate the β-phase transition of PVDF [71]. The nanoparticles obtained by carbonizing dopamine on ZnO surface not only enhanced the β-phase of PVDF, but also changed the triboelectric effect by negating the PVDF surface potential to as much as −740 mV.

Beyond that, PVDF copolymer (take PVDF-HFP as an example) is also widely used for friction materials due to its better mechanical and triboelectric properties than PVDF. Lee’s group reported a kind of stretchable and breathable friction nanofiber membrane by electrospinning PVDF-HFP and electrospraying styrene-ethylene-butylene-styrene (SEBS) elastomer microspheres [52]. According to the report, electrospraying SEBS microspheres combined with PVDF-HFP nanofibers can improve the tensile properties (~490%) and superhydrophobicity (~140°) of the fibers without affecting the triboelectric property. On this basis, Lee’s group studied further by adding perovskite nanoparticles (Cs_3_Bi_2_Br_9_) to PVDF-HFP nanofibers as a highly efficient electron acceptor and local nucleating agent for polymer chain crystallization (Figure 6b) [53]. Notably, the good energy level matching between PVDF-HFP and perovskite nanoparticles of nanofiber composites increases electron transfer efficiency and friction performance. Due to its mechanical strength and flexibility, the produced nanofiber film can detect human movements and gather mechanical energy as individuals walk and bend their arms (Figure 6c). Good washing stability and output charge density made it a highly efficient energy harvesting device for wearable self-powered sensors (Figure 6d). In addition to the triboelectric effect, the functionalization of fiber films to satisfy actual application prospects is also important. For example, the electrosprayed fluorinated CNTs can not only enhance the triboelectric property [54], but also endow the fiber film with a self-cleaning function (Figure 6e). The prepared flexible TENG sensor for agricultural detection can be attached to plant leaves without sacrificing the inherent physiological activities of plants due to its waterproof and breathable ability (Figure 6f), and can be used as a sustainable power source for wireless plant sensors to realize real-time monitoring of plant health status by collecting energy from the raindrop.

**Figure 7:**
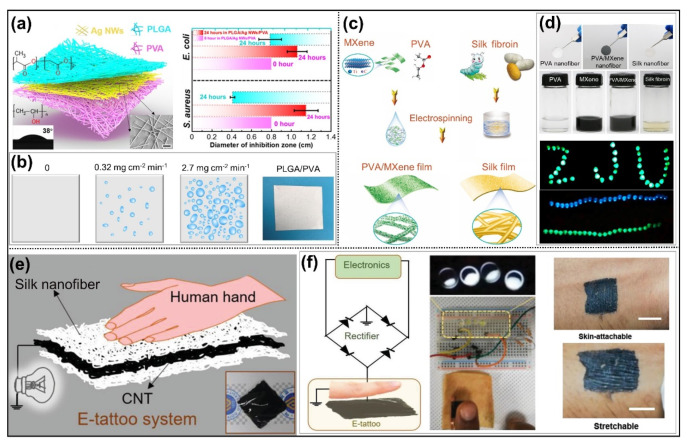
Triboelectric materials based on biodegradable nanofibers applied for energy harvesting. (**a**) A biodegradable and antibacterial PLGA/PVA nanofiber film for TENG. (**b**) The schematic illustration of sweat on the PLGA/PVA e-skin. Reprinted with permission from Ref. [55]. Copyright 2020 Science. (**c**) The preparation of silk and PVA/Mxene-based nanofiber film for TENG. (**d**) The obtained nanofiber films yield high power density. Reprinted with permission from Ref. [56]. Copyright 2019 Elsevier. (**e**) A sandwich structured silk nanofiber film for TENG. (**f**) The flexible silk film can be adhered directly to the human skin. Reprinted with permission from Ref. [57]. Copyright 2021 Wiley-VCH.

Although friction materials such as PVDF-HFP, PTFE, and PDMS have good friction qualities, their poor biocompatibility and degradability prevent their future research and deployment. Researchers have become more interested in biodegradable materials, such as polylactic acid (PLA), polycaprolactone (PCL), polyvinyl alcohol (PVA), silk fiber, and other natural polymers in recent years [72,73]. Pan et al. used electrospinning PLA nanofibers as friction material with a maximum instantaneous output charge density of 5 W m^−2^ [74]. It is worth noting that this material can be completely degraded in water for 40 days. In biomedical applications such as skin sensors and implanted electronic devices, biodegradable electronic sensors provide superior benefits over traditional chemical fiber materials [75]. Materials that are biocompatible and biodegradable can significantly lessen the harm caused to the human body. Moreover, because the skin sensor adhering to the skin will not induce inflammation, fiber-based sensors implanted into human tissues can avoid rejection responses and can be destroyed and absorbed naturally by tissues over time [76]. In this way, biodegradable material would greatly decrease the harm caused by the secondary removal of electronic devices in the human body and reduce the cost. Wang et al. group reported a nanofiber-based TENG with breathable, biodegradable, antibacterial, and energy harvesting properties when bonded to human skin (Figure 7a) [55]. The electrospun electronic skin may create a balance between antibacterial and biodegradable properties by altering the concentration of Ag nanowires and the mix of PVA and PLGA. The bactericidal effectiveness against Escherichia coli and Staphylococcus aureus is 54% and 88%, respectively, and the majority of degradation may be achieved in 21 days. The produced TENG with a 3D micro-nano porous structure is capable of transferring heat and moisture to provide wearer comfort. Due to the porosity nature of electrospinning nanofiber, this electronic skin’s (120 mm s^−1^) air permeability is greater than that of commercial denim. Figure 7b depicts the amount of sweat rate, which can still play triboelectric performance under the maximum sweat amount (about 2.7 mg cm^−2^ min^−1^).

Silk, on the other hand, being a natural biological material, is utilized in a variety of sectors, including biosensing, optics, biomedicine, and energy storage [72]. Silk fibroin and sericin, which are rich in an abundance of amino, amine, and other electron-donating groups, are the primary components of silk [77]. Therefore, silk is an ideal material for TENG application as a tribopositive layer to enhance the output performance of bio-TENG. Electrospinning is the primary method for producing silk nanofiber membranes. Prior to that, Bombyx mori silkworm cocoons were sliced into flakes and degummed to remove the sericin on the surface of the fibroin. This method can regenerate silk fibers at the nanoscale scale and has the benefits of high application and tunable physical structure. Jiang et al. [56] developed the fiber-based TENG by electrospinning silk fibroin fiber as the electron donor and PVA/Mxene as the electron acceptor (Figure 7c). With a maximum instantaneous peak power density of 1087.6 mW m^−2^, the two nanofiber friction materials with differing electronegativity exhibit exceptional electrical qualities (Figure 7d). Compared to the mismatch between the triboelectric layer and the electrode layer, silk nanofiber may be employed as an electrode that is compatible with the silk triboelectric layer, therefore reducing the incompatibility issue to some extent. Figure 7e shows a sandwich structured silk-based electronic skin tattoo mainly composed of silk nanofibers/CNT flexible electrode [57]. The electronic tattoo can be utilized for skin energy collection and tactile recognition in human-machine interfaces due to the triboelectric feature of silk thread. When the tattoo comes into contact with bare skin, the maximum power density is 6 mW m^−2^, which is sufficient for lighting diodes and stopwatches. Additionally, the silk electrode was protected by silk fiber membrane and could adhere freely to human skin by van der Waals force and withstand stretching (Figure 7f).

## 4. Summary and Outlook

As a green energy harvesting technology, TENG is distinguished by its small weight, simple form, and high material adaptability. Particularly, its application potential lies within the realms of portable energy and intelligent wearables. However, the lack of a flexible and stable substrate severely limits its further development. Hence, the integration of TENG with fabric/fiber materials and structures is crucial to the future of smart wearable self-powered sensors. Due to the advanced processing technology, TENG can be designed into different textile structures (1D, 2D, 3D) with a variety of functionalities. Fabric/fiber-based TENG has been extensively explored, including textile fabric and fibers, wet spinning micron fibers, and electrospinning nanofibers, as this study demonstrates. Different weaving techniques maximize the combined benefits of fabric/fiber-based TENG. On the one hand, nanogenerators may be easily integrated into fabric or fiber materials, granting the fabric or fiber the capacity to gather mechanical energy and perform self-powered sensing. On the other hand, the nanogenerators’ excellent wear resistance, permeability, wear comfort, structural flexibility, and low cost creates good conditions for their widespread use.

### 4.1. Potential Applications

#### 4.1.1. Health Care Monitoring

Benefits to their inherent flexibility and good wearing comfort, the fabric/fiber based TENG can be comfortably attach on the human body as a self-powered sensor to monitor human parameters, such as limb movement, pulse and other health conditions. Doctors can monitor a person’s health remotely via wireless transmission. Moreover, the good permeability and wash ability of fabric/fiber-based sensor make it more adaptable in real-world applications.

#### 4.1.2. Smart Sports System

TENGs can be made by various materials, including wood, fibers and polymers, which are mostly adopted materials in sports facilities and sports clothes. Thus, TENG devices can be installed on sports facilities or directly made into them. In this way, the triggered electric signal can be recorded and analysed to assess the exercise parameters. Moreover, the physiological signals of human body can also be detected in real time together with the electric signal of sports facilities, which can get a more accurate data for evaluating the exercise condition.

### 4.2. Future Challenges

Compared with traditional rigid electronic devices, the integration of TENG with fabric/fiber-based materials are advantageous for its good wear comfort. Although fabric/fiber structured TENGs can be prepared by the above discussed techniques, it should be mentioned that several challenges must be resolved before their commercial application.

1. First, the preparation of TENG through spinning and weaving requires specific textiles. Not all fabrics can be used as triboelectric materials, because of their weak triboelectricity or wear resistance; besides, the spinnability also pose a great challenge to many triboelectric materials for their unsatisfied mechanical strength in twist and draw process. Moreover, the adding of electrodes to textiles may alter their wearability and contribute additional complexity to the weaving process, which requires more study on the spinning and weaving technics
2. As for wet spinning, one of the primary drawbacks is that if a single fiber breaks during the spinning process, the continuity of the spinning process will be disrupted, meaning that the process requirements are quite stringent in comparison to other methods. More than that, the obtained fiber-shaped electrode still needs further coatings of triboelectric layers on the surface, which may cause interfacial adhesion problems. It is quite important to improve the continuity of spinning and enhance the interfacial adhesion between tribomaterial and fiber electrode for the future commercial application.
3. Electrospinning is still adopted by researchers thanks to its simplicity, low cost, and extensive selection of triboelectric materials. However, large-scale production inevitably restricts other uses. The technological and process strategies are currently in the research phase.

Overall, despite the fact that the state-of-the-art fabric/fiber structured TENG has a few flaws, it considerably advances the development of wearable self-powered sensors and makes it feasible to create green flexible wearable power sources. Furthermore, fabric/fiber structured TENGs are also expected to applied in other fields, such as food detection, wastewater treatment and electro-catalysis [78,79,80,81]. It is hoped that fabric/fiber structured TENGs would continue to provide scientists with fresh insights and provide further research results.

## Figures and Tables

**Table 1:** Summary and comparison of fabric/fiber-based TENGs.

Electrodes	Triboelectric Materials	Structures and Substrates	Main Methods	Power Outputs	Ref.
Silver	PDMS	Core-shell type; carbon fiber	Coating	2.5 μW	[20]
Conductive yarn	PTFE yarn	Core-shell type; yarns; fabric	Fancy twisting	3.36 µA; 180.06 V.	[24]
Conductive fiber	PVDF/PAN	Core-shell structure; yarns; fabric	Electrospinning; woven	40.8 V, 0.705 μA cm^–2^	[25]
Silver paste coated cotton	PTFE	Textile fabric	Coating	343.19 mW m^−2^	[35]
Conductive fabric	FDTS, PVDF-HFP	Textile fabric	Dipping	0.11 mW, 22 V	[45]
Silver-coated nylon yarns	PVDF	Core-shell type; woven fabric	Weaving	1008 mW m^−2^	[46]
Conductive yarn	PI	3D honeycomb structure; woven fabric	Hollow spindle fancy twisting	73.55 µW m^−1^	[47]
Silver-plated nylon	PDMS	3D braiding structure; Braided yarn, axial yarn	Multiaxial yarn winding	26 W m^−3^	[48]
CNT, MXene, Au	PDMS	Shell-core structure; PU fiber	Wet spinning; dipping	6 V	[49]
Hydrogel electrode	PMA	Shell-core structure; woven hydrogel fabric	Wet spinning; coating	~88 mW m^−2^	[28]
Ni; Al	PVDF/G; PA 6	Nanofiber film	Electrospinning; spin-coating	~130.2 W m^−2^	[44]
Conductive fabric	Pt-PVDF	Nanofiber film	Electrospinning	22 µW cm^−2^	[50]
Copper	PVDF/Mxene; PA 6/6	Nanofiber film	Electrospinning	11.213 Wm^−2^	[51]
Liquid metal; Ag flakes	PVDF-HFP	Nanofiber film	Electrospinning; electrospraying	219.66 mW m^−2^	[52]
Silver	PVDF-HFP/ Cs_3_Bi_2_Br_9_	Nanofiber film	Electrospinning; electrospraying	2.34 Wm^−2^	[53]
CNT electrode	PVDF-HFP; F-CNT	Nanofiber film	Electrospinning; electrospraying	330.6 μW cm^−2^	[54]
AgNWs	PLGA	Sandwich structure; nanofiber film	Electrospinning	130 mW m^−2^	[55]
Al	Silk fibroin; PVA/MXene	Nanofiber film	Electrospinning	1087.6 mW m^−2^	[56]
CNT	Silk fiber	Sandwich structure; nanofiber film	Electrospinning	6 mW m^−2^	[57]

## Data Availability

Not applicable.
